# Mutagenesis identifies the critical amino acid residues of human endonuclease G involved in catalysis, magnesium coordination, and substrate specificity

**DOI:** 10.1186/1423-0127-16-6

**Published:** 2009-01-15

**Authors:** Shih-Lu Wu, Chia-Cheng Li, Jaw-Chyun Chen, Yi-Jin Chen, Ching-Ting Lin, Tin-Yun Ho, Chien-Yun Hsiang

**Affiliations:** 1 Department of Biochemistry, China Medical University, Taichung 40402, Taiwan; 2 Molecular Biology Laboratory, Graduate Institute of Chinese Medical Science, China Medical University, Taichung 40402, Taiwan; 3 Department of Microbiology, China Medical University, Taichung 40402, Taiwan

## Abstract

**Background:**

Endonuclease G (EndoG), a member of DNA/RNA nonspecific ββα-Me-finger nucleases, is involved in apoptosis and normal cellular proliferation. In this study, we analyzed the critical amino acid residues of EndoG and proposed the catalytic mechanism of EndoG.

**Methods:**

To identify the critical amino acid residues of human EndoG, we replaced the conserved histidine, asparagine, and arginine residues with alanine. The catalytic efficacies of *Escherichia coli*-expressed EndoG variants were further analyzed by kinetic studies.

**Results:**

Diethyl pyrocarbonate modification assay revealed that histidine residues were involved in EndoG activity. His-141, Asn-163, and Asn-172 in the H-N-H motif of EndoG were critical for catalysis and substrate specificity. H141A mutant required a higher magnesium concentration to achieve its activity, suggesting the unique role of His-141 in both catalysis and magnesium coordination. Furthermore, an additional catalytic residue (Asn-251) and an additional metal ion binding site (Glu-271) of human EndoG were identified.

**Conclusion:**

Based on the mutational analysis and homology modeling, we proposed that human EndoG shared a similar catalytic mechanism with nuclease A from *Anabaena*.

## Background

Endonuclease G (EndoG) belongs to the large family of DNA/RNA non-specific ββα-Me-finger nucleases [1]. *In vitro *studies indicated that EndoG is involved in several biological functions. For examples, EndoG is capable of processing primers for mitochondrial DNA replication [2]. EndoG is also an apoptotic protein that releases from mitochondria during apoptotic process and serves as an alternative pathway to cause genomic DNA fragmentation [3-5]. Moreover, EndoG initiates herpes simplex virus type 1 (HSV-1) recombination event by cleaving the HSV-1 *a *sequence [6]. It is also required for normal cellular proliferation [7].

In mammals, EndoG is synthesized as a propeptide in the cytoplasm and imported into mitochondria through a process mediated by its amino-terminal mitochondrial-targeting sequences [2,8]. EndoG preferentially cleaves DNA at double-stranded (dG)_n_·(dC)_n _and at single-stranded (dC)_n _tracts, producing 5'-phosphomonoester ends [9]. The addition of EndoG to isolated nucleus first induces higher order chromatin cleavage into large DNA fragments, followed by inter- and intranucleosomal DNA cleavages [10]. Although the cleavage patterns of EndoG on plasmid and chromatin have been identified, the critical amino acid residues of human EndoG remain to be clarified.

A few nuclease structures have been solved so far. For examples, nuclease A (NucA) from *Anabaena*, nuclease from *Serratia*, E-group colicins from *Escherichia coli *(*E. coli*), I-*Ppo*I from *Physarum polycephalum*, and Vvn from *Vibrio vulnificus *are sugar-nonspecific nucleases involved in host defense [11]. The active sites of these nucleases display a similar ββα-Me-finger topology [12]. The critical amino acid residues involved in nuclease activities have also been well known. For examples, histidine residues (His-124 in NucA, His-89 in *Serratia *nuclease, His-103 in colicin E9, His-98 in I-*Ppo*I, and His-80 in Vvn) act as general bases to active water molecules for the nucleophilic attacks on the phosphorus atoms [13-17]. Arginine residues (Arg-93 in NucA, Arg-57 in *Serratia *nuclease, Arg-5 in colicin E9, Arg-61 in I-*Ppo*I, and Arg-99 in Vvn) donate hydrogen bonds to nonbridging oxygens of the scissile phosphoryl groups and stabilize the product 5' phosphate [15,16,18-20]. Asparagine residues (Asn-155 in NucA, Asn-119 in *Serratia *nuclease, Asn-119 in I-*Ppo*I, and Asn-127 in Vvn) bind to the essential magnesium ions, which interact with the 3'-oxygen leaving groups [14,16,21,22]. In this study, we analyzed the roles of conserved histidine, asparagine, and arginine residues in the catalysis, magnesium coordination, and substrate specificity of human EndoG. Previous study indicated that H-N-N motif of bovine EndoG is essential for catalysis [1]. Herein we demonstrated that the H-N-N motif (His-141, Asn-163, Asn-172) of human EndoG was critical not only for catalysis but also for substrate specificity. His-141 was involved in magnesium coordination, suggesting the unique role of His-141 in both the catalysis and the magnesium coordination. In addition to H-N-N motif, the asparagine and glutamic acid residues near the C terminus of EndoG were identified to play a role in the catalysis and magnesium binding, respectively.

## Methods

### Cloning of human EndoG cDNA

To isolate the human EndoG cDNA, total RNA was extracted from HeLa cells, reverse transcribed by SuperScript™ III (Invitrogen, Carlsbad, CA, USA), and amplified for 35 cycles with P3 (5'-CGGGATCCGCCGAGTTGCCCCCTGTGCC-3') and M1 (5'-CGGAATTCTCACTTACTGCCCGCCGTGATGG-3') primers. The 755-bp EndoG cDNA fragment (Δ1–48) was inserted into the *Bam*H I and *Eco*R I sites of histidine-tagged expression vector pET-28c(+) (Novagen, Madison, WI, USA) to create the pET-EndoG. The plasmid DNA created in this study was confirmed as an in-frame construction by sequencing.

### Expression and purification of recombinant human EndoG

Recombinant human EndoG was expressed in *E. coli *BL21(DE3)pLysS strain by transforming the pET-EndoG to produce an N-terminal fusion with six histidine residues. The protein was purified as described previously with slight modification [23,24]. Briefly, cells were induced by isopropyl-β-D-thiogalactopyranoside. EndoG was then purified by nickel-affinity chromatography, with 8 M urea present through out the procedure. The protein in the final column eluate was pooled, renatured by sequential dialysis, and stored at -70°C until further analysis. Protein was analyzed by sodium dodecyl sulfate (SDS)-polyacrylamide gel electrophoresis and quantified with a Bradford assay (Bio-Rad, Hercules, CA, USA).

### Nuclease activity assay

Plasmid pUC18 dsDNA, preparing with the Qiagen plasmid midi kit (Qiagen, Valencia, CA, USA), contained mainly supercoiled and a small amount of open circular DNA. For nuclease activity, 0.1 μg of pUC18 dsDNA or *Eco*R I-linearized pUC18 dsDNA was mixed with 0.1 pmol purified human EndoG in EndoG buffer (20 mM Tris-HCl, 1 mM MgCl_2_, 0.5 mM dithiothreitol, pH 7.5) and incubated at 37°C for 5 min. The reaction was then stopped by the addition of stop solution (25% glycerol, 0.5% SDS, 0.05% bromophenol blue, 50 mM EDTA), and the resulting products were analyzed by electrophoresis on 1.2% agarose gels.

### Chemical modification and hydroxylamine restoration assay

The chemical modification was performed as described previously [25]. Briefly, EndoG (0.2 pmol) was mixed with various amounts of diethyl pyrocarbonate (DEPC) (Sigma, St Louis, MO, USA) in 50 mM potassium phosphate buffer (pH 6.0) and incubated at 25°C for 30 min. The reaction was then stopped by adding 100 mM imidazole (pH 6.0) to a final concentration of 1 mM. The residual activity was determined by nuclease activity assay. For hydroxylamine restoration assay, DEPC-modified EndoG was mixed with hydroxylamine to a final concentration of 20 mM and incubated at 4°C for 5 h. The residual nuclease activity was determined under the standard assay condition. DEPC used in this study was freshly diluted with absolute ethanol, and the ethanol concentration in the reaction mixture didn't exceed 2.5% (v/v).

### Site-directed mutagenesis

Site-directed mutagenesis was performed as described previously [25]. Uracil-containing ssDNA was prepared by transforming pET-EndoG into *E. coli *CJ236 strain, which lost its deoxyuridine triphosphate nucleotidohydrolase and uracil glycosylase activities. Uracil-containing ssDNA (0.3 pmol) was annealed with 6 pmol of 5'-kinase primer in annealing buffer (20 mM Tris-HCl, 2 mM MgCl_2_, 50 mM NaCl, pH 8.0). The second-strand DNA was then synthesized by the addition of 4 μl of 10× synthesis buffer (4 mM deoxyribonucleotides, 175 mM Tris-HCl, 37.5 mM MgCl_2_, 5 mM dithiothreitol, 7.5 mM ATP, pH 8.0), 3 units of T4 DNA ligase and 1 unit of T4 DNA polymerase, followed by sequential incubations on ice for 5 min, at 25°C for 5 min, and at 37°C for 90 min. The dsDNA was then transformed into *E. coli *NM522 strain to destroy the uracil-containing strand by uracil glycosylase activity and to allow the mutated strand to be amplified. The primers for the constructions of EndoG mutants are shown in Table 1.

**Table 1:** DNA oligonucleotides for the constructions of human EndoG mutants.

Primer name	Primer sequence^a^
R110A	5'-GCACTCTCG**AGC**GTCGCCGTCGCCGCGGAGACG-3'
R139A	5'-CAGGTGGCC**GGC**GTCGAAGCCACTGCCGCGG-3'
H141A	5'-GGCCAG**GGC**CCCGCGGTCGAAGCCACTGCC-3'
H141D	5'-GGCCAG**ATC**TCCGCGGTCGAAGCCACTGCCGC-3'
N163A	5'-GGGGCGCTAC**AGC**GCTCAGGTAGAACGTGTCGTCC-3'
N163K	5'-CACCTGGGGCGCGAC**CTT**GCTCAGGTAGAAC-3'
N172A	5'-CATTCTG**CGC**AAGGTGGGGCACCTGGGGCGCG-3'
R184A	5'-TCAAGCT**TGC**GCTATATTTCTCCAGGTTGTTCC-3'
H228A	5'-TTGAAGAA**TGC**TGTGGGCACTGCCACGTGG-3'
H228D	5'-GAAGAA**GTC**TGTAGGCACTGCCACGTGGTTCTTGCC-3'
N251A	5'-AGGTGC**TGC**AGGCATCACGTAGGTGCGGAGC-3'
E271A	5'-CGAAGCCCG**AGC**AATGCTCTCGATGGGCACCAGG-3'
R272A	5'-CAGCCCTGAGGC**CGC**CTCAATGCTCTCGATGGGCACC-3'

^a ^Bold sequences indicate codon changes

### Kinetic analysis

Cleavage kinetics was carried out by using various concentrations of pUC18 dsDNA substrate and constant amounts of EndoG [26]. Reactions were initiated by combining reaction buffer, substrate, and enzyme in that order. Samples were mixed and incubated at 37°C for 3 min. The products and substrates were then separated by agarose gel electrophoresis, and the intensities of products and substrates on the gel were measured by the Gel-Pro^® ^Analyzer (Media Cybernetics, Inc., Silver Spring, MD, USA). The initial velocity was calculated by using the equation v = {I1/(I0+0.5I1)t} × [substrate], where t = time in seconds, I1 = product intensity, and I0 = substrate concentration. Vmax and *K*m values were calculated by directly fitting the data to the Michaelis-Menten equation, and *k*cat and *k*cat/*K*m were then derived.

### Sequence-specific cleavage assay

Sequence-specific cleavage assay was performed as described previously with modification [6]. The plasmid DNA pKJH20, containing the HSV-1 *a *sequence, was kindly provided by Ke-Jung Huang (Department of Biochemistry, Beckman Center, Stanford University). The pKJH20 DNA was cleaved by *Eco*R I and *Xba *I to generate 2.8-kb and 1.6-kb fragments. Recombinant human EndoG was mixed with 0.2 μg of *Eco*R I/*Xba *I-treated pKJH20 in EndoG buffer containing 15 mM spermidine and incubated at 37°C for various periods. The reactions were then stopped by the addition of stop solution, and the resulting products were analyzed by electrophoresis on 1.2% agarose gels.

### Protein structure prediction

The structure of human EndoG was modeled using the NucA from *Anabaena *(PDB code 1ZM8) as the reference protein. Protein structure was generated via the GeneSilico metaserver gateway [27]. 'FRankenstein's monster' approach was applied to refinement of the EndoG structure [28].

## Results

### Chemical modification of human EndoG

**Figure 1:**
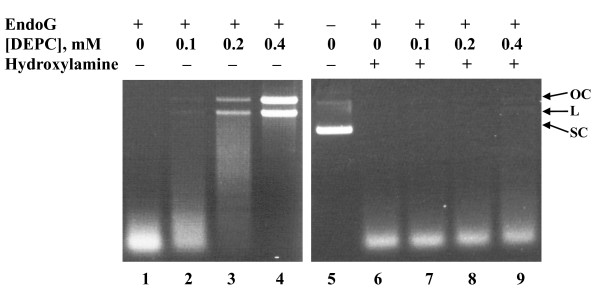
**Chemical modification of human EndoG**. EndoG (0.2 pmol) was mixed with various amounts of DPEC and incubated at 25°C for 30 min. The residual activity was analyzed by standard assay conditions (lanes 1 to 4). An assay for restoration with hydroxylamine was performed by incubating 20 mM hydroxylamine and DEPC-treated EndoG together at 4°C for 5 h. The residual activity was then analyzed (lanes 6 to 9). Lane 5 represents the reaction performed in the absence of EndoG. Arrowheads denote the different topological forms of pUC18 plasmids. OC: open circular; L: linear; SC: supercoiled.

Histidine residue has been implicated in the active site of several nucleases, including colicin E9, *Serratia *nuclease, I-*Ppo*I, Vvn, and viral deoxyribonuclease (DNase) [13,25,29,30]. In order to determine whether the histidine residue was also responsible for the catalytic activity of human EndoG, we treated EndoG with various amounts of DEPC. The residual catalytic activities of DEPC-modified EndoG were then determined under standard assay conditions. DEPC can modify different nucleophiles (such as amine, alcohol, thiols, imidazole, and guanido group), producing the carbethoxyl derivatives. At pH 6.0, DEPC is mostly specific for histidine; however, it also reacts to a smaller extent with lysine. The modified residues could be further differentiated by hydroxylamine restoration assay. Lysine-modified enzymes cannot recover their activities in the presence of hydroxylamine, whereas histidine-modified enzymes retrieve their functions after the treatment of hydroxylamine [31]. Carbethoxylation of EndoG by DEPC resulted in a loss of enzyme activity, and the inactivation was dose-dependent (Figure 1). However, treatment of DEPC-modified EndoG by hydroxylamine restored the lost catalytic activity. These findings suggested that histidine residues were involved in the catalytic activity of human EndoG.

### Enzyme kinetics of human EndoG variants

**Figure 2:**
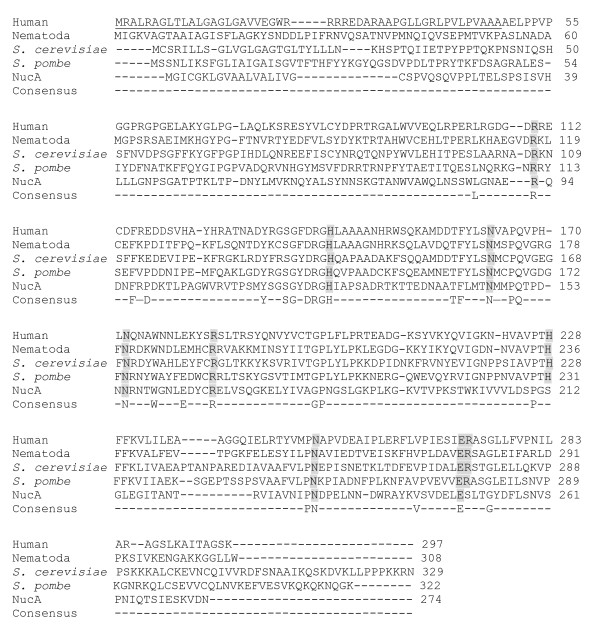
**Multiple alignment of EndoG homologs with NucA**. Amino acid sequences of EndoG homologs, including human, nematode (*Caenorhabditis elegans*), budding yeast (*Saccharomyces cerevisiae*), and fission yeast (*Schizosacharamyces pombe*), were compared with those of NucA by PileUp program. Residues that are identical in all nucleases are shown at bottom. The location of signal peptide is underlined. The amino acid residues mutated in this study are shaded in grey.

In addition to histidine residues, we also analyzed the roles of asparagine, arginine, and glutamic acid residues in EndoG activity. Asparagine and arginine are essential for catalysis in various nucleases [32-34]. Glutamic acid has also been implicated in the magnesium binding of nucleases [22,35]. By multiple alignment of EndoG homologs from human, nematode, and yeast, we found that histidine residues at positions 141 and 228, asparagine residues at positions 163, 172 and 251, arginine residues at positions 110, 139, 184 and 272, and glutamic acid at position 271 of human EndoG were highly conserved (Figure 2). These conserved amino acid residues were then replaced with alanine, lysine, or aspartic acid residue to generate 14 EndoG mutants. The mutants were then expressed and purified to homogeneity, and the enzyme kinetics of EndoG mutants was analyzed under Michaelis-Menten conditions.

**Figure 3:**
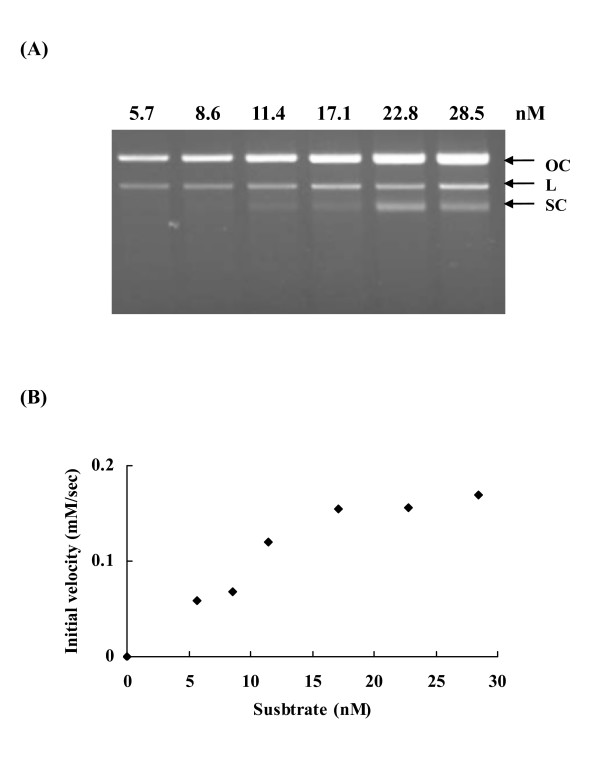
**Kinetic analysis of human EndoG**. (A) Electrophoresis analysis. EndoG (0.05 pmol) was mixed with various amounts of supercoiled pUC18 dsDNA and incubated at 37°C for 3 min. The resulting products were then analyzed by 1.2% agarose gels. Arrowheads denote the different topological forms of pUC18 plasmids. OC: open circular; L: linear; SC: supercoiled. (B) Initial velocity of DNA cleavage as a function of substrate concentration. Steady-state kinetic parameters were determined by curve fitting of these values to the Michaelis-Menten equation.

Figure 3 shows that the cleavage products (open circular and linear forms) were generated by EndoG. To determine the Michaelis constants (*K*m) and catalytic-centre activity (*k*cat) values of EndoG, nuclease assays were carried out using various concentrations of substrates, a constant amount of enzyme and gel electrophoresis, and the initial cleavage rates were measured by quantifying cleavage products. The *K*m and *k*cat values of wild-type EndoG derived from these experiments were 18.72 ± 1.84 nM and 0.07 ± 0.01 S^-1^, respectively.

A comparison of kinetic parameters for EndoG wild type and mutants is shown in Table 2. The activities of R110A and E271A mutants could not be determined accurately, as they aggregated with supercoiled pUC18 dsDNA. Replacement of His-141 and Asn-163 with alanine and lysine, respectively, resulted in a slight decrease in *K*m. However, a dramatic reduction in *K*m was observed when the conserved arginine residues at positions 139, 184, and 272 were substituted by alanine. Mutation at Asn-251 also lead to a markedly decrease in *K*m. These findings indicated that the dissociations of the Michaelis complex between mutants (R139A, R184A, N251A, and R272A) and DNA substrate were smaller than that of wild-type EndoG.

**Table 2:** Steady-state kinetic data for human EndoG variants.

EndoG variants	*K*m (nM)^a^	*k*cat (S^-1^)^a^	*k*cat/*K*m (S·nM^-1^)^a^
Wild type	18.72 ± 1.84	0.07 ± 0.01	3.58 × 10^-3 ^± 3.11 × 10^-4^
R110A	n.d.	n.d.	n.d.
R139A	1.01 ± 0.03	2.77 × 10^-5 ^± 4.44 × 10^-7^	2.75 × 10^-5 ^± 1.47 × 10^-6^
H141A	12.93 ± 2.17	2.48 × 10^-6 ^± 4.34 × 10^-7^	1.96 × 10^-7 ^± 4.78 × 10^-8^
H141D	1.94 ± 0.05	9.68 × 10^-6 ^± 2.59 × 10^-13^	5.03 × 10^-6 ^± 1.72 × 10^-7^
N163A	8.51 ± 2.28	7.52 × 10^-6 ^± 1.41 × 10^-6^	9.05 × 10^-7 ^± 7.67 × 10^-8^
N163K	12.10 ± 2.03	2.35 × 10^-6 ^± 1.85 × 10^-7^	1.99 × 10^-7 ^± 1.61 × 10^-8^
N172A	8.75 ± 0.13	3.77 × 10^-5 ^± 4.26 × 10^-8^	4.31 × 10^-6 ^± 5.95 × 10^-8^
R184A	1.53 ± 0.02	7.68 × 10^-3 ^± 3.27 × 10^-10^	5.03 × 10^-4 ^± 1.91 × 10^-5^
H228A	6.01 ± 1.88	5.34 × 10^-3 ^± 2.71 × 10^-3^	8.61 × 10^-4 ^± 1.81 × 10^-4^
H228D	5.70 ± 0.33	4.74 × 10^-4 ^± 1.78 × 10^-5^	8.33 × 10^-5 ^± 1.75 × 10^-6^
N251A	3.13 ± 0.37	3.15 × 10^-6 ^± 5.68 × 10^-14^	1.02 × 10^-6 ^± 6.64 × 10^-8^
E271A	n.d.	n.d.	n.d.
R272A	2.30 ± 0.20	3.67 × 10^-3 ^± 9.23 × 10^-5^	1.61 × 10^-3 ^± 9.96 × 10^-5^
H141A/H228A	8.06 ± 1.92	1.18 × 10^-7 ^± 1.26 × 10^-8^	1.48 × 10^-8 ^± 1.98 × 10^-9^

^a ^Data are presented as mean ± standard error of triplicate assays.

n.d., not determined.

Mutations at His-141, Asn-163, and Asn-251 showed drastically reduced activities (< 0.01%), suggesting that these amino acid residues were critical for catalysis. A large decrease in activity (~0.1%) was also observed when the asparagine at position 172 was substituted by alanine. Less dramatic effects (> 10%) were observed when His-228, Arg-184, and Arg-272 were replaced with alanine. Most of the mutants were mainly affected in their *k*cat, while H141D and N251A exhibited a decrease in both *k*cat and *K*m. N172A was more affected in its *k*cat than *K*m. These findings suggested that His-141, Asn-163, Asn-172, and Asn-251 of human EndoG were involved in catalysis.

### Magnesium requirements of human EndoG variants

**Figure 4:**
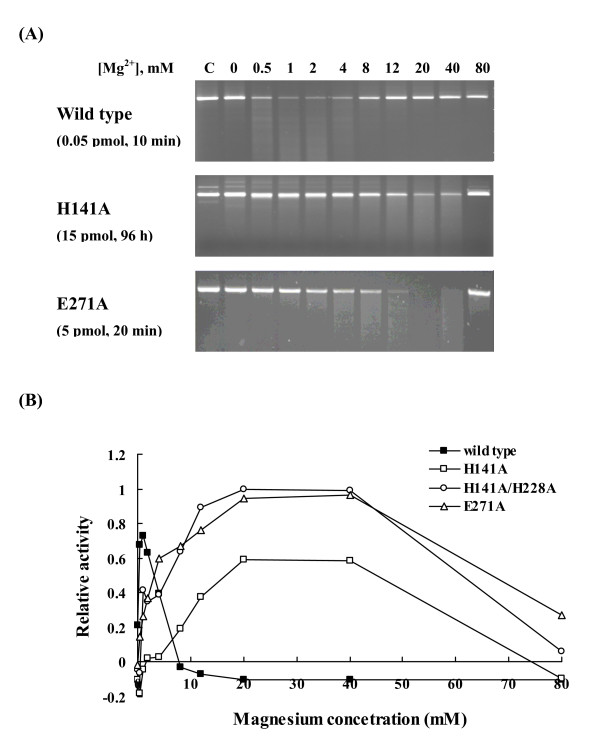
**Magnesium requirements of human EndoG variants**. (A) Electrophoresis analysis. EndoG variants were mixed with 0.1 μg of linear pUC18 dsDNA in EndoG buffer containing various amounts of magnesium. The reaction mixtures were incubated at 37°C for indicated periods. The resulting products were analyzed by 1.2% agarose gels. Lane C represents the reaction performed in the absence of EndoG. (B) Densitometric analysis. Activities are given as relative values with respect to the maximum activity for each variant.

We investigated the magnesium requirements of EndoG wild type and mutants as described previously [13]. The maximal catalytic activity of wild-type EndoG would be achieved at magnesium concentration of 1 mM (Figure 4). Moreover, the enzyme activity of wild-type EndoG was inhibited at a higher magnesium concentration. All the EndoG mutants shared similar profiles with wild type (data not shown). However, H141A and E271A achieved the maximal activities at a 20-fold higher magnesium concentration (20 mM) than wild-type EndoG. The optimal magnesium concentration of H141A/H228A double mutant was almost the same as that of H141A. These mutants had lower affinities for the magnesium ion cofactor than the wild-type EndoG, suggesting that magnesium ion might be coordinated directly by His-141 and Glu-271 of human EndoG.

### Cleavage specificities of human EndoG variants

**Figure 5:**
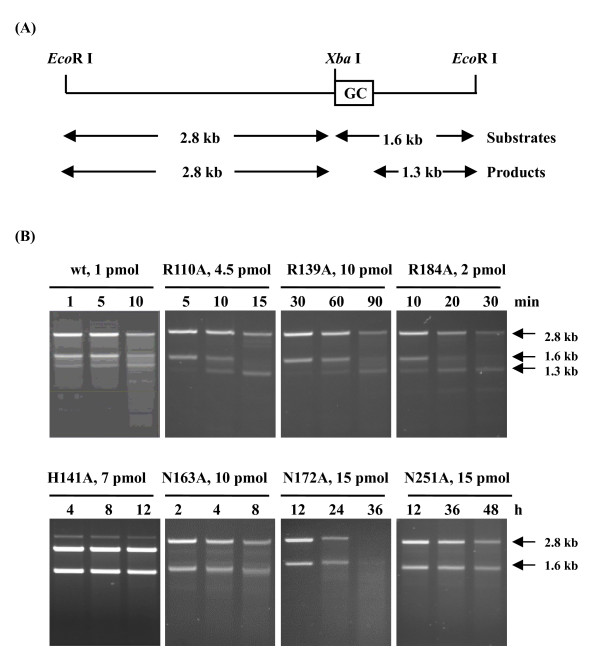
**Cleavage specificities of human EndoG variants**. (A)Sequence-specific cleavage assay. The structure of *Eco*R I-linearized pKJH20 DNA is shown at the top, with the open box designating the GC-rich sequence region (0.3 kb). *Xba *I site is located 1.6 kb from the *Eco*R I site at the 3' end of the linear pKJH20 DNA. In the cleavage assay, pKJH20 was cleaved by *Eco*R I and *Xba *I to generate the 2.8-kb and 1.6-kb substrates. Enzymes cleaving within the GC-rich sequence are expected to generate both the 2.8-kb and the 1.3-kb product fragments. (B) Electrophoresis analysis. The reaction mixtures containing 0.2 μg *Eco*R I/*Xba *I-treated pKJH20 and EndoG variants were incubated at 37°C for various periods. The resulting products were analyzed by 1.2% agarose gels. Arrowheads denote the substrate and product fragments.

EndoG is highly specific for (dG)_n_·(dC)_n _tracts in DNA, generating single strand cleavages within the dG·dC homopolymer pair [9]. We further analyzed the roles of conserved amino acid residues in the cleavage specificity of human EndoG. Sequence-specific cleavage assay was carried out by using pKJH20 as the substrate. Plasmid pKJH20 was derived from pBluescript II SK (+) by inserting a 340-bp HSV-1 *a *sequence-containing fragment into the *Bam*H I site and a 1.24-kb kanamycin cassette into the *Pst *I site. The *a *sequence is a GC-rich (85%) fragment, containing many strings of dG_n _(n = 4–8) [6]. Plasmid pKJH20 was cleaved by *Eco*R I and *Xba *I to generate the 2.8-kb and 1.6-kb substrates, where the GC-rich fragment is located within the 1.6-kb fragment (Figure 5A). When the enzyme cleaved DNA within the GC-rich fragment, the 1.6-kb substrate fragment would be cleaved to generate the 1.3-kb product fragment. As shown in Figure 5B, wild-type EndoG initially cleaved the 1.6-kb fragment to generate the 1.3-kb fragment. With prolonging the digestion time, EndoG cleaved both fragments (2.8 kb and 1.6 kb) to generate diffuse bands. Mutations at arginine residues displayed the similar cleavage patterns with wild type. R110A, R139A, and R184A preferentially cleaved the 1.6-kb fragment. When the incubation period was extended, the 1.6-kb fragment was totally digested to generate the 1.3-kb fragment, and both fragments (2.8 kb and 1.3 kb) were consequently degraded to generate diffuse bands. In contrast to these mutants, H141A, N163A, N172A, and N251A cleaved both fragments equally. With extending the digestion time, both fragments were degraded to low-molecular-weight-fragments. These findings suggested that His-141, Asn-163, Asn-172, and Asn-251 were critical for substrate specificity, while Arg-110, Arg-139, and Arg-184 might not be involved in the cleavage specificity.

## Discussion

**Figure 6:**
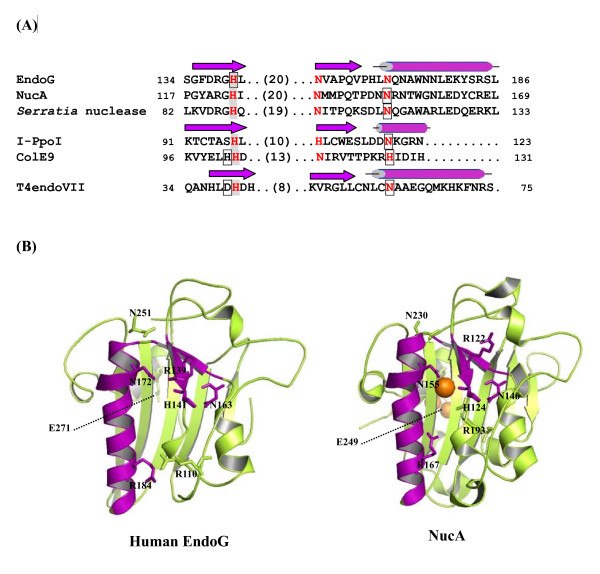
**Sequence alignment and structure of H-N-H nucleases**. (A) Multiple alignment of human EndoG H-N-N motif with H-N-H nucleases. Amino acid sequences of human EndoG, NucA, *Serratia *nuclease, ColE9, I-*Ppo*I, and T4 endoVII were aligned. The amino acid residues representing the H-N-H motif are highlighted in red. The two β-strands and the α-helix of H-N-H motif are indicated by arrows and tube, respectively. The amino acid residues acting as general bases are highlighted in grey. Boxed residues represent the amino acid residues involved in magnesium coordination. (B) Comparison of the modeled structure of human EndoG and the experimentally solved structure of NucA (PDB code 1ZM8). ββα-Me-finger motif is highlighted in purple. Amino acid residues involved in catalysis, magnesium coordination, and substrate specificity are indicated and labeled.

In this study, we demonstrated the roles of conserved histidine, arginine, and asparagine residues in catalysis, magnesium coordination, and substrate specificity of human EndoG. The primary sequence analysis revealed that EndoG contained the DRGH prosite motif, which is a characteristic of highly active, divalent metal ion-dependent, non-specific nucleases (Figure 6A) [22]. The site-directed mutagenesis analysis demonstrated that H-N-N motif (His-141, Asn-163, and Asn-172) of human EndoG were critical for catalysis and substrate specificity. The secondary structure analysis revealed that EndoG contained an active site with the ββα-Me-finger fold, which is the characteristic of many H-N-H superfamily endonucleases (Figure 6A) [34]. Based on the results of our analysis, we reported herein the biochemical evidence for human EndoG that belongs to the H-N-H superfamily and exhibits a catalytic motif based on the ββα-Me-finger fold.

The H-N-H nucleases include heterogeneous group of enzymes with diverse functions but with similar active sites. A few examples are: sugar nonspecific nucleases such as NucA and *Serratia *nuclease, nonspecific DNases such as *E. coli *colicin E9, and homing endonucleases such as I-*Ppo*I [17,21,22,36]. A similar ββα-Me topology has been revealed in the active site regions of these nucleases [12]. Moreover, the H-N-H motif, characterized by the presence of a conserved Asn/His residue flanked by conserved His and His/Asn residues at some distance, is presented in these endonucleases [17,31,36]. The histidine residue in the ββα-Me finger is ideally positioned to act as a general base to activate a water molecule for the nucleophilic attack on the phosphorous atom [22]. By results of mutational analysis, we revealed that His-141 was responsible for catalysis of human EndoG. His-141 was also highly conserved among these nucleases (NucA, His-124; *Serratia *nuclease, His-89; colicin E9, His-103; I-*Ppo*I, His-98) [13-15,17]. These findings indicated that His-141 of human EndoG would act as a general base.

Magnesium, the most abundant divalent metal ion in mammalian cells, plays structural and catalytic roles in many cellular processes [37]. Magnesium ion functions as a cofactor of proteins involved in DNA replication and repair pathways. It is required for activity and fidelity of DNA polymerase. It is also required for the activities of nucleases, such as apurinic/apyrimidinic endonuclease, MutH, RNase A, DNase I, and viral DNase [23,38-41]. Additionally, magnesium ions are not only required for the phosphoryl-transfer reaction, they also play a role in substrate binding [42]. The enzyme activities of EndoG and *Serratia *nuclease were inhibited at a higher magnesium concentration [13], suggesting that excessive amount of magnesium ion might interfere the nuclease-DNA interaction and result in the inhibition of enzymatic activities. The active site of the H-N-H endonucleases supplies one or two magnesium ligands as identified by crystal structure analysis: Asn-155 in NucA, Asn-119 in *Serratia *nuclease, His-102 and His-127 in colicin E9, and Asn-119 in I-*Ppo*I [14,17,21,22]. Traditionally, enzymes that utilize magnesium are known to contain metal-binding sites that are created by acidic residues [43-45]. However, there is precedent for the binding of magnesium by histidine in several enzymes. For examples, His-607 and His-643 of phosphodiesterase-5 act as direct participants in coordinating the magnesium required for catalysis [33]. His-102 and His-127 of colicin E9 are included in the coordination shell of a magnesium ion in the colicin E9 active site [17]. Previous study suggested that Asn-174 of bovine EndoG, corresponding to Asn-172 of human EndoG, is a putative magnesium ligand [1]. By results of mutagenesis, we demonstrated that His-141 instead of Asn-172 might be coordinated with magnesium because H141A mutant required a higher magnesium concentration to achieve the maximal activity. These findings suggested the unique role of His-141 in both catalysis and magnesium binding of human EndoG. In addition to His-141, an additional residue (Glu-271) located near the C terminus of human EndoG was also coordinated with magnesium ion. A second metal binding site (glutamic acid) located near the C terminus of the protein was present in human EndoG (Glu-271) and NucA (Glu-249) but not in other H-N-H endonucleases [22]. These findings suggested the closest similarity between EndoG and NucA.

Human EndoG participates in the HSV-1 genomic inversion by cleaving the *a *sequence [6]. The recombinant human EndoG preferentially cleaved the GC-rich fragment, also indicating that EndoG preferentially cleaved at the GC-rich sequence. However, the sequence preferences of EndoG mutants were disappeared; H141A, N163A, N172A, and N251A cleaved both fragments to generate low-molecular-weight fragments. These results suggested that His-141, Asn-163, Asn-172, and Asn-251 were required not only for catalysis but also for sequence specificity of human EndoG. Previous study proposed that arginine residues are critical for DNA binding of bovine EndoG [1]. However, the cleavage specificities of R110A, R139A, and R184A were similar as that of wild type. These results suggested that arginine residues at positions 110, 139, and 184 of human EndoG might be involved in the DNA substrate interactions but not in the cleavage site determination.

**Figure 7:**
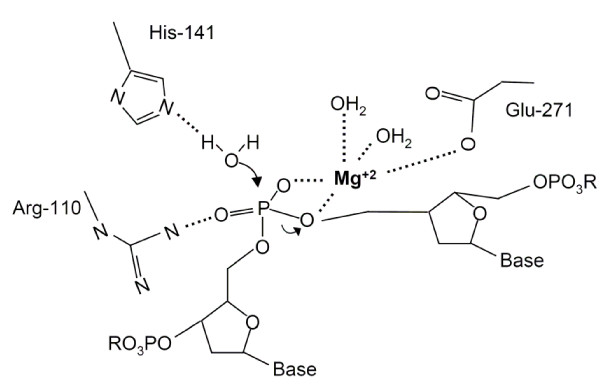
**Proposed catalytic mechanism of EndoG**. His-141 acts as a general base to activate a water molecule, generating a nucleophile that attacks the phosphodiester bond. Arg-110 and magnesium ion stabilize the transition state.

Mutational analysis indicated that H-N-N motif (His-141, Asn-163, Asn-172) of human EndoG was essential for catalysis. N251A mutant lost its enzyme activity, indicating that Asn-251 was also involved in catalysis. Moreover, His-141 and Glu-271 were critical for magnesium coordination. EndoG and NucA share ~24.4% sequence identity (~39.5% similarity), which is most pronounced in the active site region (Figure 2). Moreover, EndoG shares similar enzyme activities and biological functions with NucA. For examples, both proteins are divalent metal ion-dependent nonspecific nucleases, which are characterized by the DRGH PROSITE motif [46]. They also participate in host defense [22]. Therefore, we build a homology model of the three-dimensional structure of human EndoG based on the crystal structure of NucA (Figure 6B). A good structural superposition for human EndoG and NucA could be achieved. Three arginine residues, Arg-110, Arg-139, and Arg-184, were distributed on a line. Mutation on these arginine residues resulted in the decreased *K*m, suggesting that arginine residues were involved in DNA binding. A rigid V-shaped architecture of human EndoG formed the catalytic site, where His-141, Asn-163, and Asn-172 were located within the cleft. Based on the mutational analysis and homology modeling, we proposed that the V-shaped cleft of human EndoG might be involved in DNA cleavage, DNA binding, and substrate recognition. Additionally, it seems very likely that EndoG follows a mechanism that is similar to that of NucA. In this model, His-141 acts as the general base that generates a hydroxyl ion for a nucleophilic attack on the scissile phosphodiester bond by activating a water molecule (Figure 7). The side-chain nitrogen of Arg-110 donates hydrogen bond to nonbridging oxygen of the scissile phosphoryl group to stabilize the cleaved DNA product. Magnesium ion, which was coordinated by His-141 and Glu-271, also interacted with a nonbridging oxygen of the scissile phosphoryl group and stabilize the oxyanion leaving group. It is noticed that an additional catalytic residue, Asn-251, and the additional magnesium coordinated site, Glu-271, were potentially located far from the cleft. How the Asn-251 and Glu-271 cooperated with H-N-N motif and participated in the DNA catalysis remained to be further clarified.

## Conclusion

In conclusion, the roles of conserved histidine, arginine, and asparagine residues in catalytic, magnesium coordination, and substrate specificity of human EndoG were analyzed. The H-N-N motif (His-141, Asn-163, Asn-172) of human EndoG was critical for catalysis and substrate specificity. His-141 was also essential for magnesium coordination. An additional catalytic residue (Asn-251) and an additional magnesium ligand (Glu-271) of human EndoG were identified. Based on the mutational analysis and homology modeling, we speculated that human EndoG shared a similar catalytic mechanism with NucA from *Anabaena*.

## Competing interests

The authors declare that they have no competing interests.

## Authors' contributions

SLW participated in the design of the study, analyzed the data, and helped to draft the manuscript. CCL carried out the mutagenesis, kinetics analysis, and protein structure prediction. JCC carried out the protein structure prediction and drafted the manuscript. YJC and CTL carried out the expression and sequence-specific cleavage analysis. TYH and CYH conceived of the study, participated in its design and coordination, and helped to draft the manuscript. All authors read and approved the final manuscript.
